# Tie1 contributes to the development of ovarian hyperstimulation syndrome under the regulation of EGR1 in granulosa cells

**DOI:** 10.1038/s12276-021-00722-8

**Published:** 2022-01-25

**Authors:** Lihua Sun, Hui Tian, Songguo Xue, Hongjuan Ye, Xue Xue, Rongxiang Wang, Yu Liu, Caixia Zhang, Qiuju Chen, Shaorong Gao

**Affiliations:** 1 School of Life Sciences and Technology, Tongji University, Shanghai, China; 2 Department of Reproductive Medicine Center, Shanghai East Hospital, Tongji University School of Medicine, Shanghai, China; 3 Department of Assisted Reproduction, Shanghai Ninth People’s Hospital, Shanghai JiaoTong University School of Medicine, Shanghai, China

**Keywords:** Mechanisms of disease, Endocrine reproductive disorders

## Abstract

The expression of tyrosine kinase with immunoglobulin-like and epidermal growth factor-like domains 1 (Tie1), a transmembrane protein expressed almost exclusively by endothelial cells, has been reported in granulosa cells. However, its significance in ovarian hyperstimulation syndrome (OHSS), which can occur after the injection of gonadotropins in infertile women undergoing controlled ovarian stimulation, is unknown. Here, we report significantly increased Tie1 and vascular endothelial growth factor (VEGF) expression in cultured granulosa cells from OHSS patients, as well as ovaries from rats with experimentally established OHSS, compared to controls, with the levels of both proteins also increasing in granulosa and SVOG cells (a nontumorigenic human granulosa-lutein cell line) treated with an acute dose of human chorionic gonadotropin (hCG). Tie1 silencing abolished the hCG-induced VEGF level in SVOG cells and attenuated the progression of OHSS in rats, as determined by histological analysis. Further studies in SVOG cells revealed that the hCG-induced upregulation of Tie1 expression involved the phosphoinositide 3-kinase/protein kinase B signaling pathway. We also report that early growth response protein 1 (EGR1), whose expression was also upregulated by hCG, bound directly to the Tie1 promoter and activated its transcription. Taken together, our results indicate that Tie1 may be a therapeutic target in cases of moderate-to-severe OHSS. Further studies are needed to address its clinical relevance.

## Infertility: Ovarian hyperstimulation syndrome

Identification of a hormone-sensitive signaling pathway in a subset of ovarian cells could lead to better treatment for women experiencing adverse events associated with infertility treatment. Up to 8% of women who receive the ‘pregnancy hormone’ human chorionic gonadotropin (hCG) during fertility care will develop ovarian hyperstimulation syndrome (OHSS), a condition that can be debilitating or even fatal. Researchers led by Qinju Chen of Shanghai JiaoTong University and Shaorong Gao of Tongji University, Shanghai, China, have identified links between blood vessel function and risk of hCG-associated complications. Working with both human cells and animal models, they showed a strong connection between hCG exposure and expression of a signaling protein associated with blood vessels in certain ovarian cells. Targeted inactivation of this protein prevented OHSS in rats, suggesting it could offer a drug target for at-risk women.

## Introduction

In the first week of pregnancy, cells surrounding the newly fertilized egg produce human chorionic gonadotropin (hCG), the so-called “pregnancy hormone”, which subsequently stimulates the production of progesterone to maintain pregnancy and establish the placenta. In infertile women undergoing controlled ovarian stimulation, hCG is administered at extremely high doses to stimulate the release of eggs from mature follicles^1^. However, these women are at an increased risk of developing ovarian hyperstimulation syndrome (OHSS), with approximately 3–8% of women experiencing moderate-to-severe symptoms within weeks of hCG injection^2,3^. In OHSS, blood vessels respond negatively to hCG, thereby causing them to leak fluids into the ovaries (thus resulting in their enlargement), abdomen, and sometimes chest^2,4^. The risk of developing OHSS is further increased in women receiving multiple hCG injections or in those achieving pregnancy. Symptoms can be mild, moderate, or severe, and the severity of symptoms dictates the treatment approach. Severe symptoms can include persistent nausea and vomiting, shortness of breath, abdominal and chest pain, pleural effusion, blood clots, kidney failure, and, in some cases, even death^5^. Furthermore, several cases of spontaneous OHSS have also been reported, and these women have a mutation in the follicle-stimulating hormone receptor (*FSHR*) gene, which displays an increased sensitivity to hCG^6,7^.

Endothelial cells that constitute the inner surfaces of blood vessels are important regulators of vasculogenesis, angiogenesis, vascular remodeling and permeability, and inflammation^8,9^, and the involvement of vascular endothelial growth factor (VEGF), a signaling protein that binds to VEGF receptors-1 (VEGFR1) and -2 (VEGFR2), in these processes has been unequivocally established^10^. Interestingly, a previous study implicated VEGF as the primary inducer of vascular permeability in patients with OHSS, and VEGF expression has been reported in human granulosa cells, where its level has been shown to increase significantly in response to hCG administration^11^. Other studies have confirmed that an elevated serum VEGF level following hCG administration can trigger the development of OHSS in women undergoing in vitro fertilization (IVF)^12,13^, indicating that the serum VEGF level can predict the risk of OHSS and that the odds of developing OHSS may be reduced by inactivating VEGF receptors and inhibiting the VEGF signaling pathway^14^. At the cellular level, hCG has been reported to trigger OHSS by upregulating VEGF expression in luteinized granulosa cells^15^. Other studies have demonstrated VEGF expression in peripheral blood mononuclear cells from women with OHSS^16^, suggesting a role for circulating immune cells in the pathophysiology of OHSS, as well as in granulosa cells, where the VEGF level has been shown to significantly increase in response to hCG. Taken together, these findings indicate that VEGF is a critical regulator of endothelial cell function.

The VEGF signaling pathway is not the only vascular tissue-specific tyrosine kinase cascade known to be involved in the pathophysiology of OHSS; studies have also identified angiopoietin (Ang)-tyrosine kinase with immunoglobulin-like and epidermal growth factor-like domain signaling pathways and implicated it in endothelial cell function and dysfunction^17,18^. Tie1 and Tie2, which constitute the Tie family of receptors, are expressed almost exclusively by endothelial cells^19,20^ Tie2 functions as a receptor for the Ang family of ligands (Ang1–4), whereas Tie1 functions as an orphan receptor; it is activated indirectly by ANGs through its interaction with Tie2. Several studies have reported Tie1 and Tie2 expression in granulosa and theca cells, which has prompted investigators to examine the roles of these receptors in the development, maturation, and release of follicles in various species, including humans^21–23^. In cows, for example, a microarray study revealed that the Tie1 level was significantly upregulated during the early luteal phase^24^, suggesting that Tie1 might be involved in the release of eggs from mature follicles. Other studies have demonstrated that the binding of Ang proteins to Tie2 activates the PI3K/AKT signaling pathway through the phosphorylation of AKT^25,26^.

The goals of this study were twofold: (i) to examine the role of Tie1 in the pathophysiology of OHSS and (ii) to elucidate its mechanism of action using in vitro and in vivo models of the disease, which included human follicular fluid specimens and an experimentally induced rat model of the disease, as well as human primary granulosa cells and SVOG cells, a human granulosa–lutein cell line, both of which were treated with a high dose of hCG to mimic the cellular events occurring during IVF. Here, we report that VEGF and Tie1 levels were elevated in granulosa cells from women with OHSS and rats with established OHSS, as well as in cells treated with hCG, confirming earlier findings reported by others. We also establish the relationship between VEGF and Ang-Tie signaling pathways, as determined by Tie1 knockdown experiments in vitro and in vivo, and show that the mechanism of action also involves the PI3K/Akt cascade, indicating that the regulation of the ovarian vasculature is more complex than initially believed. Taken together, our results indicate that Tie1 may be a therapeutic target in cases of severe OHSS.

## Materials and methods

### Patients and collection of follicular fluid

Infertile patients undergoing oocyte retrieval were enrolled at Shanghai East Hospital. All patients provided written informed consent before study commencement. Patients diagnosed with OHSS (*n* = 23) were divided into the mild OHSS group (*n* = 21) and moderate OHSS group (*n* = 2). The diagnosis of OHSS was based on clinical practice guidelines published in 2016. Mild hyperstimulation was defined as bilateral ovarian enlargement and an ovarian diameter <8 cm, accompanied by abdominal bloating and mild abdominal pain. Moderate hyperstimulation was defined based on the patient’s assessment of discomfort, abdominal bloating, ascites, or nausea, as well as hematopoietic cell transplantation >0.41 and an ovarian diameter of 8–12 cm. The non-OHSS (control) group consisted of age-matched patients (*n* = 8). Follicular fluid was collected at the time of oocyte retrieval and centrifuged at 2000*g* for 10 min. The Institutional Review Board of Shanghai East Hospital approved the procedures.

### Controlled ovarian hyperstimulation protocol

A recently revised COH protocol, known as progestin-primed ovarian stimulation, was used. On Day 3 of the menstrual cycle and onward, patients received 150–225 IU of human menopausal gonadotropin (hMG; Anhui Fengyuan Pharmaceutical Co., China) and 10 mg of medroxyprogesterone acetate (MPA; Shanghai Xinyi Pharmaceutical Co., China). The initial dose of hMG was adjusted according to the patient’s body mass index, and the subsequent doses of hMG were adjusted according to the follicular size and serum hormone levels during treatment cycles. When at least one dominant follicle was 20 mm in diameter or three follicles were >18 mm in diameter, oocyte maturation was induced with 0.1 mg triptorelin (Decapeptyl; Ferring Pharmaceuticals, Germany) and 1000 IU human chorionic gonadotropin (hCG; Lizhu Pharmaceutical Trading Co., China). Transvaginal ultrasound-guided oocyte retrieval was performed at 36–38 h after induction.

### Isolation, culture, and treatment of human granulosa cells

Granulosa cells were collected from follicular fluid specimens of five OHSS patients at the time of oocyte retrieval, purified by density gradient centrifugation, and cultured in DMEM/F12 (Gibco, Shanghai, China) supplemented with 10% fetal bovine serum (FBS) in a humidified atmosphere of 37 °C and 5% [v/v] CO_2_. Cells were seeded and stimulated with 10 IU/mL of hCG (Lizhu, Guangdong, China) at 60–70% confluence for 6–24 h and subsequently harvested for RNA and protein extraction.

### Culture and treatment of the SVOG cell line

SVOG cells were cultured in DMEM/F12 supplemented with 10% [w/v] FBS in a humidified atmosphere of 37 °C and 5% [v/v] CO_2_. For inhibitor treatment, SVOG cells were pretreated with 20 μM of LY294002 (Selleck, Houston, TX, USA), a potent nonselective inhibitor of PI3Ks, for 1 h, followed by treatment with 10 IU/mL hCG for 24 h. Cells were subsequently harvested for RNA and protein extraction.

### Measurement of the VEGF level

VEGF levels in follicular fluid specimens were quantified using the human VEGF Quantikine Enzyme-linked Immunosorbent Assay (ELISA) Kit (cat. no. EK183-02; MultiSciences/LiankeBio, Hangzhou, China) according to the manufacturer’s instructions. Absorbance readings were obtained with a BioTek EPOCH microplate reader (Winooski, VT, USA).

### Reverse transcription-quantitative polymerase chain reaction (RT-qPCR)

To examine the levels of target genes in granulosa and SVOG cells exposed to different treatments as described herein, total RNA was isolated using TRIzol Reagent (Invitrogen, Carlsbad, CA, USA) according to the manufacturer’s instructions, and its concentration and purity were determined with a NanoDrop2000 spectrophotometer (Thermo Fisher, Waltham, MA, USA). Thereafter, 2 μg of RNA was reverse transcribed using the PrimeScript RT Reagent Kit (Takara, Dalian, China). SYBR Premix Ex *Taq* II (Takara) and gene-specific primers were employed for RT-qPCR, which was performed using the LightCycler 96 system (Roche). The primers used for RT-qPCR were as follows: Tie1 (forward, 5′-AAGCAGACAGACGTGATCTGG-3′; reverse 5′-GCACGATGAGCCGAAAGAAG-3′), VEGF (forward, 5′-CTTGCCTTGCTGCTCTACCT-3′; reverse, 5′-GCAGTAGCTGCGCTGATAGA-3′), and *GAPDH* (forward, 5′-GATGCCCCCATGTTCGTCAT-3′; reverse, 5′-TCTTCTGGGT GGCAGTGATG-3′). The specificity of each reaction was determined by melting curve analysis and agarose gel electrophoresis of the amplified target genes. The relative mRNA level was determined using the comparative Ct method. Water and total RNA served as negative controls for RT-qPCR. *GAPDH* was used for the normalization of target gene levels.

### Western blotting

To examine the levels of target proteins in granulosa and SVOG cells exposed to different treatments, cells were rinsed in phosphate-buffered saline and lysed in radioimmunoprecipitation assay buffer (Beyotime, Beijing, China) freshly supplemented with protease inhibitor cocktail (Roche, Shanghai, China). Protein concentrations were determined using the Bicinchoninic Acid Protein Assay Kit (Thermo Fisher). Equivalent amounts of total protein (30 μg) were separated on 10% sodium dodecyl sulfate-polyacrylamide gel electrophoresis gels and electrophoretically transferred to polyvinylidene fluoride membranes. Membranes were blocked with 5% [w/v] nonfat milk in TBST for 1 h and subsequently incubated in antibodies against Tie1 (1:1000; Abcam, Cambridge, UK), VEGF (1:1000; Santa Cruz Biotechnology, Santa Cruz, CA, USA), or EGR1 (1:1000; Cell Signaling Technology, Danvers, MA, USA) overnight at 4 °C. Thereafter, membranes were washed briefly with TBST, incubated with species-compatible horseradish peroxidase-conjugated secondary antibodies (1:1000; Santa Cruz Biotechnology) for 1 h at room temperature, and then washed extensively in TBST. Immunoreactive proteins were developed with chemiluminescent reagents (Bio-Rad) and visualized with the ChemiDoc MP Imaging System (Bio-Rad). The relative intensities of target proteins were quantified by ImageJ Software (NIH, Bethesda, MD, USA). β-Actin was used for the normalization of target protein levels.

### Small interfering (si) RNA transfection and target gene silencing in vitro

Approximately, 2 × 10^5^ SVOG cells were seeded in the wells of 6-well plates and cultured for 24 h, as previously indicated. To ensure target specificity, siRNAs were titrated, and the lowest effective concentration of each siRNA was used for further studies. Thereafter, cells were transfected with human Tie1 siRNA (50 pmol; 5′-GGGAGAGGAGGUUUAUGUGAA-3′; GenePharm, Shanghai, China) or EGR1 siRNA (50 pmol; 5′-GCAUCUGCAUGCGCAACUU-3′; GenePharm) for 24 h. siRNAs were diluted in Opti-MEM I reduced-serum medium (ThermoFisher), and RNAi MAX Reagent (Invitrogen) was used for transfection according to the manufacturer’s instructions. Scrambled/nontargeting siRNAs (50 pmol; GenePharm) served as the negative controls for silencing experiments. SVOG cells were subsequently harvested for RNA and protein extraction.

### Chromatin immunoprecipitation (ChIP)

The EZ-Magna ChIP A/G Chromatin Immunoprecipitation Kit (Millipore, Billerica, MA, USA) was used for ChIP according to the manufacturer’s instructions. In brief, treated and untreated SVOG cells were crosslinked with 1% [v/v] formaldehyde for 10 min at room temperature and incubated with an antibody against EGR1 (1:50; Cell Signaling Technology) or rabbit IgG at an equivalent concentration. Thereafter, A/G magnetic beads were added, and samples were incubated at 4 °C overnight to pull down the coimmunoprecipitated DNA, which was subsequently quantified by PCR and qPCR. The primers for PCR/qPCR were as follows: Tie1 promoter (forward, 5′-ACCTCAAGTGACTCCTCCCA-3′; reverse, 5′-TGCTGGGGAGGAAGAGGAAAT-3′).

### Construction of vectors and dual-luciferase reporter assays

The *Tie1* genomic DNA sequence was downloaded from Genome Browser (http://genome.ucsc.edu/), which is hosted by the University of California at Santa Cruz, and the −2000-bp sequence upstream of the transcription start site was annotated. To identify the transcription factors potentially binding to the Tie1 promoter, we used JASPAR (http://jaspar.genereg.net/). Different fragments of the promoter region were amplified by PCR using genomic DNA extracted from SVOG cells and subsequently cloned into the restriction sites for BgIII and NheI, which lie upstream of the firefly luciferase gene in the pGL3-basic vector (Promega, Madison, WI, USA). Thereafter, SVOG cells were seeded in the wells of 24-well plates, cultured overnight as previously indicated, and cotransfected with 0.2 μg of the promoter-luciferase plasmid (−1958/+100, PGL3-Tie1–1; −1015/+100, PGL3-Tie1–2; −581/+100, PGL3-Tie1–3; −259/+100, PGL3-Tie1–4) and 0.01 μg of the pRL-TK plasmid, which served as the internal control, using Lipofectamine 2000 Reagent (Invitrogen) for 24 h. SVOG cells were then cultured in the absence or presence of 10 IU/mL hCG for 24 h. Luciferase activity was measured using the Dual-Luciferase Reporter Assay System (Promega) according to the manufacturer’s instructions, and the luciferase activity was normalized to the protein concentration of each sample.

### Establishment of OHSS in rats and target gene silencing in vivo

To induce OHSS in rats, 20-day-old female Wistar rats weighing 45–50 g were procured from the Shanghai SLAC Laboratory Animal Co., Ltd. (Shanghai, China) and housed in a specific-pathogen-free room maintained at a temperature of 22 °C, a relative humidity of 40–70%, and cycles of 12 h light:12 h dark with free access to standard rat chow and free water. Guidelines were issued by the Institutional Animal Ethics Committee of Shanghai East Hospital and followed strictly. The rats were divided into two groups as follows: (a) the control group received 0.1 mL of saline intraperitoneally on Days 22–26, and (ii) the OHSS group received 10 IU of pregnant mare serum gonadotropin for 4 consecutive days, followed by 30 IU hCG on Day 5 to induce experimental OHSS as previously described^27^. Approximately, 4 h after hCG administration, the rats were decapitated, and the ovaries were immediately removed. The right ovaries were submerged in 4% [w/v] paraformaldehyde for hematoxylin and eosin staining, and the left ovaries were frozen in liquid nitrogen for protein extraction and western blotting.

Tie1 silencing in vivo was performed as previously described^28^. In brief, siRNAs were diluted in siRNA buffer and subsequently mixed with atelocollagen using the AteloGene Local & Systemic Use Kit (Tokyo, Japan) according to the manufacturer’s instructions. The rats were anesthetized intraperitoneally with 2% [v/v] pentobarbital at 50 mg/kg body weight, and the ovarian bursa was exposed. Thereafter, 10 μL of the siRNA/atelocollagen complex containing 10 ng of siRNA was injected at the base of the oviduct using a NanoFil microinjection syringe (WPI, Sarasota, FL, USA) under a standard laboratory stereomicroscope. The incision was closed with a 4-0 Vicryl Rapide absorbable suture (Ethicon, Somerville, NJ, USA). The sequence of the rat *Tie1* siRNA was 5′-GGAGGAAGUAUAUGUGAAGAA-3′ and that of the scrambled siRNA was 5′-GGTGAGAAGTAGAGAGATATA-3′ (Thermo Fisher).

### Histological analysis

Ovaries harvested from control and experimentally induced OHSS rats, as well as those from OHSS rats subjected to Tie1 or nontargeting silencing, were used for histological analysis. Paraffin-embedded ovaries were cut into 5-μm-thick sections. Sections were deparaffinized and rehydrated, followed by staining with modified Harris hematoxylin and eosin (Thermo Fisher). The number of corpus lutea, as an indicator of recent ovulation, was counted in three cross-sections obtained through the center of the ovary and averaged. Images were acquired with a Nikon Eclipse E100 microscope (Tokyo, Japan).

### Statistical analysis

Statistical differences were determined by Student’s *t* test for comparisons between two groups and by ANOVA for comparisons among three or more groups. Statistical analysis was performed using GraphPad Prism 5.0 Software (San Diego, CA, USA). Each experiment was performed at least three independent times, with similar results obtained each time. Data were expressed as means ± SEM. Data were considered statistically significant at *P* < 0.05.

## Results

### VEGF and Tie1 levels increase significantly in granulosa cells from OHSS patients

**Fig. 1:**
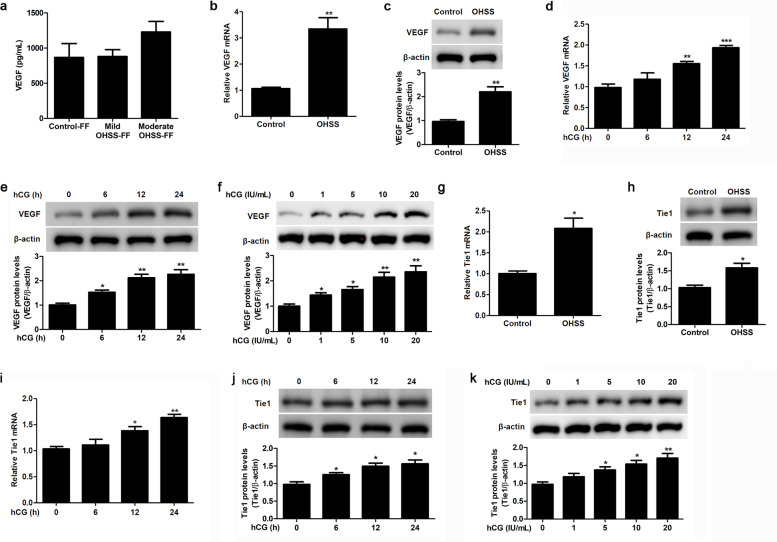
VEGF and Tie1 levels increase significantly in cultured granulosa cells from OHSS patients. **a** VEGF level in follicular fluid specimens from control (control-FF, *n* = 8), mild-OHSS (mild-OHSS-FF, *n* = 21), and moderate-OHSS patients (moderate-OHSS-FF, *n* = 2), as quantified by ELISA. **b**, **c** VEGF levels in cultured granulosa cells from control and OHSS patients, as determined by RT-qPCR (**b**) and western blotting (**c**). **d**, **e** VEGF levels in granulosa cells cultured in the absence or presence of 10 IU/mL hCG for the indicated periods of time, as determined by RT-qPCR (**d**) and western blotting (**e**). **f** VEGF levels in granulosa cells cultured in the presence of 0–20 IU/mL hCG for 24 h, as determined by western blotting. **g**, **h** Tie1 levels in cultured granulosa cells from control and OHSS patients, as determined by RT-qPCR (**g**) and western blotting (**h**). **i**, **j** Tie1 levels in granulosa cells cultured in the absence or presence of 10 IU/mL hCG for the indicated periods of time, as determined by RT-qPCR (**i**) and western blotting (**j**). **k** Tie1 levels in granulosa cells cultured in the presence of 0–20 IU/mL hCG for 24 h, as determined by western blotting. β-Actin served as the internal control. The optical density units of each target protein were normalized to β-actin and expressed as fold-change. The data from three independent experiments are expressed as arbitrary units using the mean ± SEM. ^*^P < 0.05, ^**^P < 0.01, ^***^P < 0.001.

To examine the involvement of vascular tissue-specific signaling pathways in the pathophysiology of OHSS, in which the integrity of ovarian blood vessels is compromised by hCG, thereby resulting in fluid leakage and accumulation in the abdomen and sometimes the chest^2,4^, we focused our attention on one key protein from the VEGF signaling pathway (i.e., VEGF) and one from the Ang-Tie signaling pathway (i.e., Tie1). There was no significant difference in the follicular fluid VEGF level between patients with OHSS and controls, as quantified by ELISA (Fig. 1a). In contrast, VEGF mRNA and protein levels were significantly higher in granulosa cells from OHSS patients than in cells from controls (Fig. 1b, c), and VEGF levels were increased twofold in granulosa cells from controls cultured in the presence of hCG for 24 h compared to untreated cells (Fig. 1d, e). Furthermore, as shown in Fig. 1f, VEGF levels showed a dose-dependent increase after treatment with hCG. Similarly, the Tie1 mRNA level was significantly higher in granulosa cells from OHSS patients than in cells from controls (Fig. 1g), protein expression levels also increased (Fig. 1h), and its level increased 1.5-fold in granulosa cells from controls cultured in the presence of hCG for 24 h compared to untreated cells (Fig. 1i, j). hCG treatment significantly promoted the expression of Tie1 in a dose-dependent manner (Fig. 1k).

### Tie1 silencing decreases the hCG-induced VEGF level in SVOG cells

**Fig. 2:**
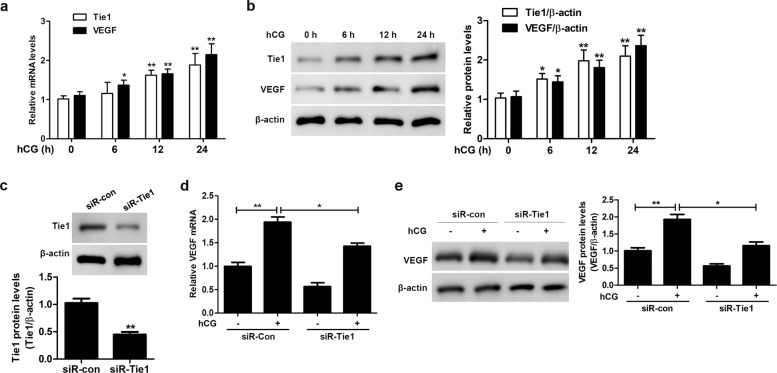
Tie1 silencing decreases the hCG-induced VEGF level in SVOG cells. **a**, **b** Tie1 and VEGF levels in SVOG cells cultured in the absence or presence of 10 IU/mL hCG for the indicated periods of time, as determined by RT-qPCR (**a**) and western blotting (**b**). **c** Tie1 levels in mock (siR-con) and Tie1 (siR-Tie1)-silenced SVOG cells, as determined by western blotting. **d**, **e** VEGF levels in mock and *Tie1*-silenced SVOG cells cultured in the absence (−) or presence (+) of 10 IU/mL hCG for 24 h, as determined by RT-qPCR (**d**) and western blotting (**e**). β-Actin served as the internal control. The optical density units of each target protein were normalized to β-actin and are expressed as fold-change. The data from three independent experiments are expressed as arbitrary units using the mean ± SEM. ^*^P < 0.05, ^**^P < 0.01.

We silenced Tie1 and quantified the VEGF level in SVOG cells cultured in the absence or presence of hCG by RT-qPCR and western blotting. Tie1 and VEGF levels increased approximately 1.5–2-fold in SVOG cells cultured in the presence of hCG for 12–24 h compared to corresponding controls, as determined by RT-qPCR and western blotting (Fig. 2a, b), indicating that gonadotropins can regulate Tie1 and VEGF mRNA and protein levels in ovarian cells. Next, we performed in vitro gene knockdown experiments (Fig. 2c). Consistent with the results shown in Fig. 2a, b, the VEGF level was significantly higher in mock-silenced (i.e., siR-con) SVOG cells cultured in the presence of hCG than in untreated mock-silenced cells (Fig. 2d, e). However, the hCG-induced increase in the VEGF level was reduced in Tie1-silenced SVOG cells compared to mock-silenced cells (Fig. 2d, e), indicative of the importance of VEGF and Ang-Tie signaling pathways in the cellular events of OHSS, which is consistent with the literature^29^.

### hCG-induced upregulation of Tie1 activates the PI3K/Akt signaling pathway in SVOG cells

**Fig. 3:**
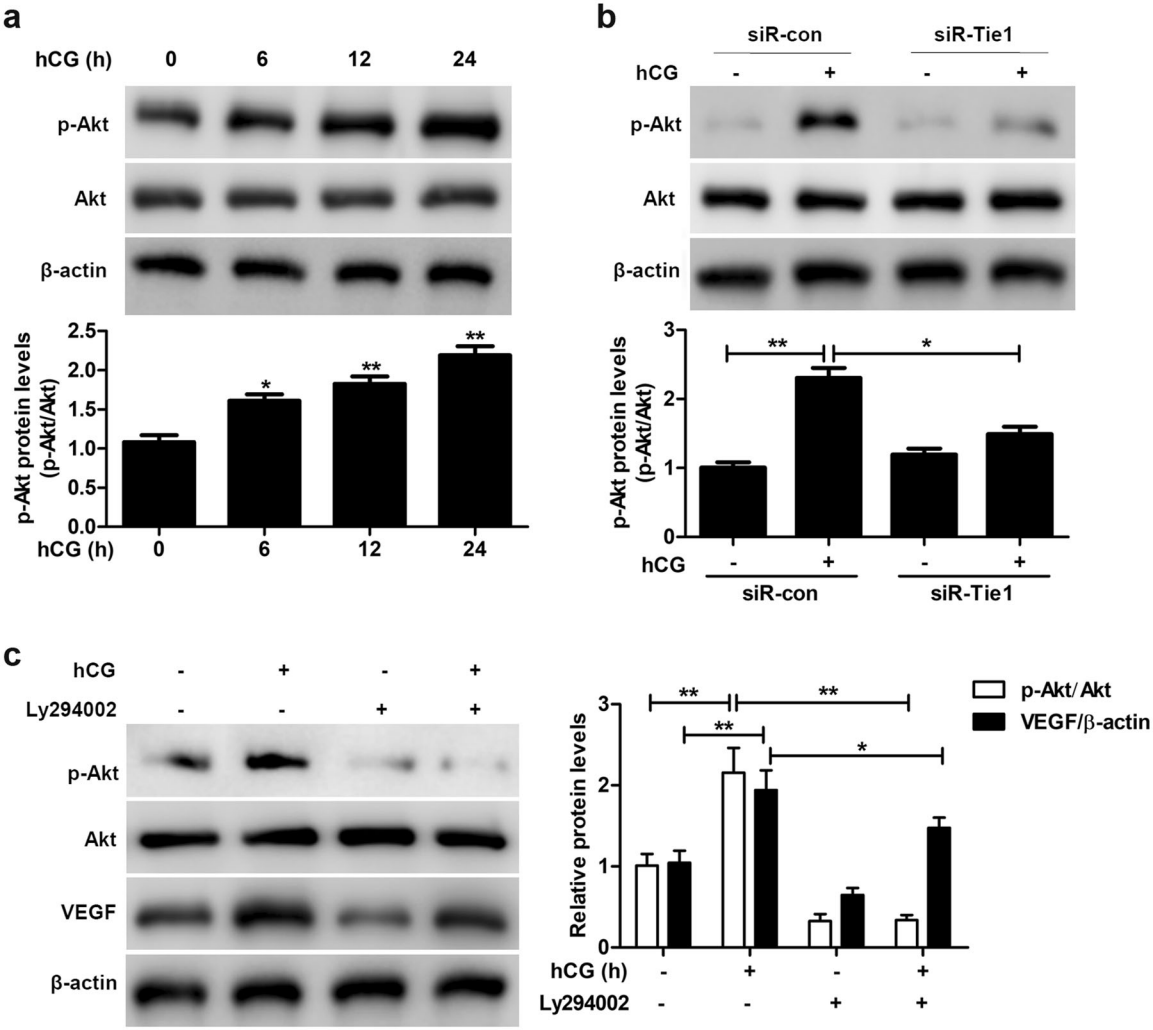
PI3K-AKT signaling is involved in the hCG-induced, Tie1-mediated increase in VEGF levels. **a** AKT and p-AKT levels in SVOG cells cultured in the absence or presence of 10 IU/mL hCG for the indicated periods of time, as determined by western blotting. **b** AKT and p-AKT levels in mock (siR-con) or Tie1 (siR-Tie1)-silenced SVOG cells cultured in the absence (−) or presence (+) of 10 IU/mL hCG for 24 h, as determined by western blotting. **c** AKT, p-AKT, and VEGF levels in SVOG cells pretreated with 20 μM LY294002 for 1 h, followed by culture in the absence or presence of 10 IU/mL hCG for 24 h, as determined by western blotting. β-Actin served as the internal control. The optical density units of each target protein were normalized to β-actin and are expressed as fold-change. The data from three independent experiments are expressed as arbitrary units using the mean ± SEM. ^*^P < 0.05, ^**^P < 0.01.

In light of the importance of the PI3K/Akt signaling pathway in the vasculature and the role of hCG in disrupting blood vessel integrity^30^, we examined the levels of AKT and phosphorylated AKT (p-AKT) in SVOG cells exposed to different treatments. The p-AKT level increased in SVOG cells cultured in the presence of hCG compared to the control (Fig. 3a). However, the hCG-induced increase in the p-AKT level was significantly reduced in Tie1-silenced cells (Fig. 3b). Moreover, the VEGF level in SVOG cells cultured in the absence or presence of hCG was suppressed by LY294002 (Fig. 3c). Taken together, these results indicate that the hCG-induced upregulation of Tie1 activates the PI3K/Akt signaling pathway in SVOG cells and that PI3K-AKT signaling may be involved in Tie1-mediated VEGF induction by hCG stimulation.

### Tie1 silencing attenuates the progression of OHSS in rats

**Fig. 4:**
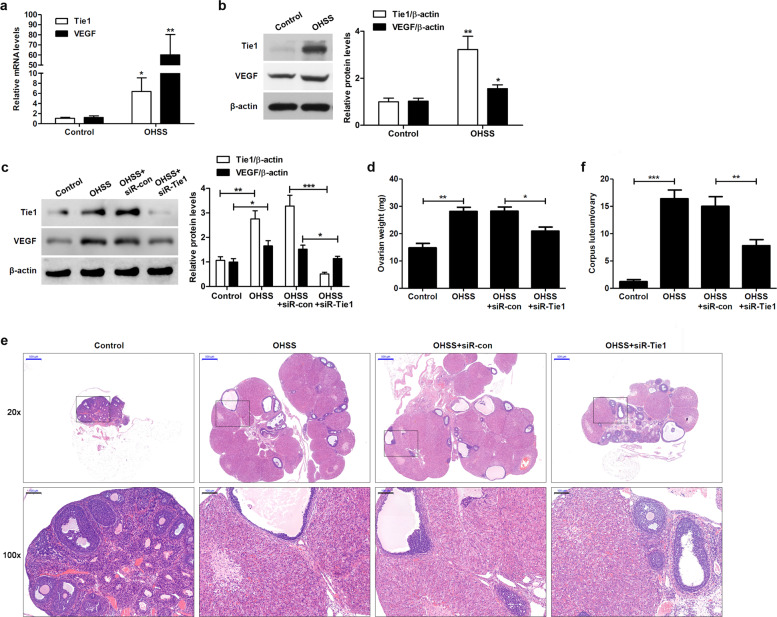
Tie1 silencing attenuates the progression of experimentally induced OHSS in rats. **a** Tie1 and VEGF levels in ovaries from control and OHSS rats, as determined by RT-qPCR. **b** Tie1 and VEGF levels in ovaries from control and OHSS rats, as determined by western blotting. **c** Tie1 and VEGF levels in ovaries from control and OHSS rats, as well as those from OHSS rats that were subjected to mock (OHSS + siR-con) or Tie1 (OHSS + siR-Tie1) silencing, as determined by western blotting. **d** Mean weights of ovaries (expressed as mg/pair) from control and OHSS rats, as well as those from OHSS rats that were subjected to mock or Tie1 silencing. **e** Representative hematoxylin and eosin-stained cross-sections of ovaries from control and OHSS rats, as well as those from rats subjected to mock or Tie1 silencing. Blue scale bar = 500 μm, black scale bar = 100 μm. **f** Number of corpus lutea (expressed as percentages) in ovaries from control and OHSS rats, as well as those from OHSS rats subjected to mock or Tie1 silencing, as determined by histological analysis (*n* = 6–8). β-Actin served as the internal control. The data from three independent experiments are expressed as arbitrary units using the mean ± SEM. ^*^P < 0.05, ^**^P < 0.01, ^***^P < 0.001.

Previous studies have implicated VEGF as a causative factor in the progression of OHSS^31,32^; however, the role of Tie1 in OHSS is unclear. To confirm the involvement of Tie1 in the pathophysiology of OHSS, we used a rat model of experimentally induced OHSS. Tie1 and VEGF levels were significantly higher in ovaries from OHSS model rats than in ovaries from corresponding controls (Fig. 4a, b). Next, we performed in vivo knockdown experiments. The VEGF level in Tie1-silenced ovaries from OHSS-model rats was significantly lower than that in mock-silenced ovaries (Fig. 4c). Furthermore, a significant increase in the weight of ovaries was observed in OHSS-model rats compared to the control, and the weight of ovaries from Tie1-silenced OHSS-model rats was significantly lower than that in mock-silenced ovaries (Fig. 4d). Additional histological analyses revealed significant increases in the size of ovaries (Fig. 4e) and the number of corpus lutea (Fig. 4f) in OHSS-model rats compared to the control, with Tie1 knockdown reducing the size of ovaries and number of corpus lutea.

### EGR1 activates transcription by directly binding to the Tie1 promoter in SVOG cells

**Fig. 5:**
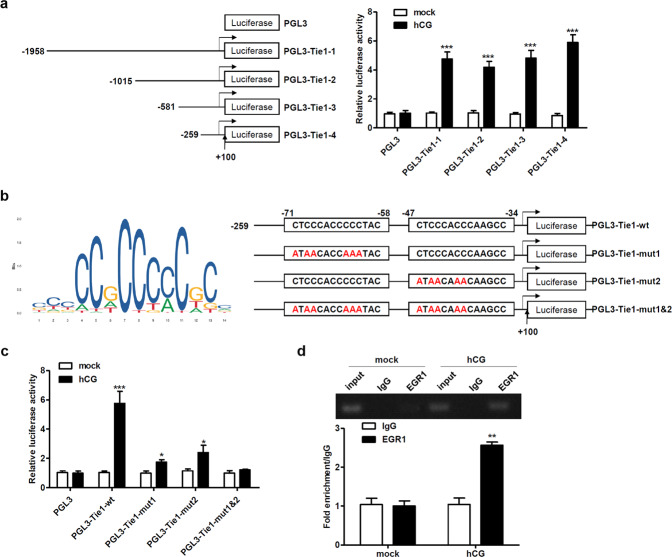
EGR1 activates transcription by directly binding to the Tie1 promoter in SVOG cells. **a** Tie1 promoter fragments were cloned upstream of the firefly luciferase reporter gene in the pGL3 vector (left). The results of luciferase reporter assays examining Tie1 promoter activity in SVOG cells (right). **b** Potential EGR1 binding site generated by JASPAR (left) and mutated sites in the Tie1 promoter (right). **c** Luciferase activity of the Tie1 promoter upon mutation of the EGR1 binding site and treatment with 10 IU/mL hCG. **d** Enrichment of EGR1 on the Tie1 promoter compared to the control (IgG) in SVOG cells treated with 10 IU/mL hCG for 24 h, as determined by ChIP assay. The results of PCR (top, 153 bp) and RT-qPCR (bottom). Input corresponds to 0.4% of the amount used per immunoprecipitation reaction. The data from three independent experiments are expressed as arbitrary units using the mean ± SEM. ^*^P < 0.05, ^**^P < 0.01, ^***^P < 0.001.

Next, we examined the transcriptional regulation of Tie1 in SVOG cells. Compared to controls, the activity of the Tie1 promoter was increased after the transfection of promoter-luciferase plasmids into SVOG cells treated with hCG (Fig. 5a). Thereafter, the potential EGR1 binding site was generated by JASPAR, and sites within the Tie1 promoter were mutated (Fig. 5b). Compared to controls, the activity of the Tie1 promoter upon mutation of the EGR1 binding site within region −71 to −58 or −47 to −34 was increased in SVOG cells treated with hCG but not upon mutation of the EGR1 binding site within both regions (Fig. 5c). To confirm the interaction of the EGR1 transcription factor with the Tie1 promoter, we performed a ChIP assay. We found increased binding of the transcription factor to the promoter in SVOG cells treated with hCG (Fig. 5d), indicating that EGR1 regulates Tie1 by binding to its promoter.

### EGR1 modulates Tie1 expression in SVOG cells

**Fig. 6:**
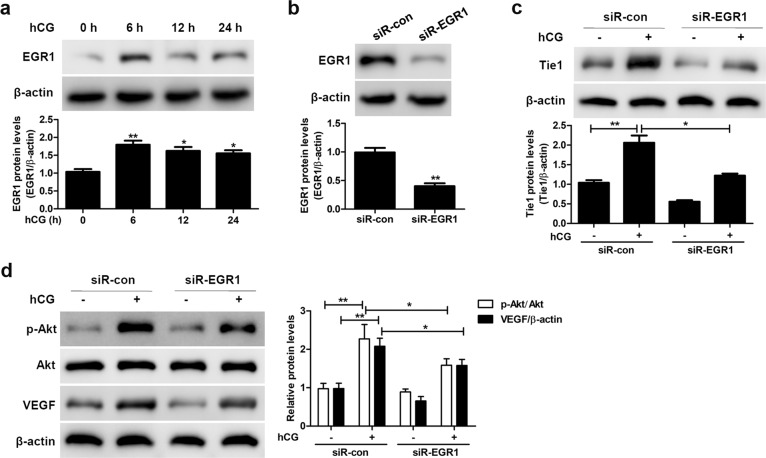
EGR1 modulates Tie1 expression in SVOG cells. **a** EGR1 level in SVOG cells cultured in the absence or presence of 10 IU/mL hCG for the indicated periods of time, as determined by western blotting. **b** EGR1 levels in mock (siR-con) or EGR1 (siR-EGR1)-silenced SVOG cells, as determined by western blotting. **c** Tie1 levels in mock or EGR1-silenced SVOG cells cultured in the absence (−) or presence (+) of 10 IU/mL hCG, as determined by western blotting. **d** AKT, p-AKT, and VEGF levels in mock and EGR1-silenced SVOG cells cultured in the absence or presence of 10 IU/mL hCG, as determined by western blotting. β-Actin served as the internal control. The data from three independent experiments are expressed as arbitrary units using the mean ± SEM. ^*^P < 0.05, ^**^P < 0.01.

Previous studies have reported an increase in the EGR1 level by various hormones, as well as upregulation in EGR1 expression in several experimental models of vascular disease;^33–36^ however, the role of EGR1 in OHSS is unknown. To confirm the involvement of EGR1 in the hCG-induced upregulation of Tie1 and activation of AKT, we silenced EGR1 and quantified the levels of Tie1, VEGF, and AKT/p-AKT in SVOG cells cultured in the absence or presence of hCG by western blotting. The EGR1 level increased approximately two to threefold in SVOG cells cultured in the presence of hCG for 6–24 h compared to the control (Fig. 6a). The efficacy of EGR1 silencing in SVOG cells was approximately 70% (Fig. 6b). The hCG-induced increase in the Tie1 level was reduced by approximately 50% in EGR1-silenced SVOG cells compared to the control (Fig. 6c). Similarly, the levels of p-AKT and VEGF were significantly higher in mock-silenced SVOG cells cultured in the presence of hCG than in untreated mock-silenced cells (Fig. 6d), whereas the levels of both proteins were lower in EGR1-silenced SVOG cells cultured in the presence of hCG (Fig. 6d). Taken together, these results indicate that the effects of hCG on the Ang-Tie signaling pathway are mediated, in part, through EGR1.

## Discussion

OHSS is a complication of controlled ovarian stimulation that aims to produce multiple ovarian follicles in the hope of increasing the number of oocytes available for in vitro fertilization^37,38^. Presently, there is no strategy to completely prevent and no protocol to successfully treat women with OHSS. Instead, women are classified according to the risk factors for OHSS, which include young age, a low body mass index, and history of the polycystic ovarian syndrome, and treated with individualized protocols, which may or may not alleviate some severe symptoms. Controlled ovarian stimulation involves the administration of a high dose of hCG, and the risk of developing OHSS is further increased in women receiving multiple hCG injections or in those achieving pregnancy. Here, we report no significant difference in the follicular fluid VEGF level between patients with OHSS and those without complications, consistent with an earlier study that showed a moderate but statistically insignificant increase in the follicular fluid VEGF level in patients with moderate or severe OHSS^13^. These discrepancies in results across studies may have been caused by variations in sample number, specimen collection and processing, and patient characteristics such as a history of previous episodes of OHSS. In contrast, VEGF and Tie1 levels were significantly higher in granulosa cells from patients with OHSS, and the levels of both proteins were further increased in granulosa cells from non-OHSS patients treated with a high dose of hCG, implicating both proteins in the pathophysiology of OHSS.

To examine the relationship between VEGF and Ang-Tie signaling pathways, we silenced Tie1 and quantified the VEGF level in SVOG cells cultured in the absence or presence of hCG. We found that VEGF and Tie1 levels were higher in treated SVOG cells than in the corresponding controls, consistent with previous studies that showed increased VEGF mRNA and protein levels in identically treated granulosa cells^11,15^. These findings prompted us to use the cell line for subsequent in vitro experiments that aimed to elucidate the roles of VEGF and Tie1. Interestingly, the hCG-induced increase in the VEGF level was attenuated in Tie1-silenced SVOG cells compared to the control, indicating that the effects of hCG on ovarian cells are mediated in part through Tie1 and that other protein are involved. Indeed, several studies have reported various growth factors, such as VEGF, to activate the PI3K/AKT signaling pathway through the phosphorylation of AKT, thereby affecting downstream proteins involved in cell survival, growth, metabolism, and angiogenesis^39^. Here, we report a significant increase in the phosphorylated AKT (p-AKT) level in SVOG cells cultured in the presence of hCG. The hCG-mediated increase in the p-AKT level, as well as that of VEGF, was eliminated after cells were treated with a nonselective PI3K inhibitor, indicating that the effects of hCG on ovarian cells are mediated through various signaling pathways. These results also revealed that the role of granulosa cells in the pathophysiology of OHSS is greater than that initially believed. It is likely that hCG induces structural changes in granulosa cells that mimic those in endothelial cells; that is, hCG may affect the function of tight junctions, thereby disrupting the permeability barrier. It is also likely that Tie1 activates a survival mechanism via PI3K signaling, as Tie1 has been reported to participate in cell growth and proliferation^40^, and *Tie1* deletion in mice reduced Tie2 phosphorylation and AKT activation^41^. However, additional studies are needed to identify the key proteins involved in these cellular events and to determine if tight junction dynamics are disrupted.

In a rat model of OHSS, we found changes in target protein levels to correlate with changes in ovarian weights, and the increase in the weight of ovaries was indicative of significant fluid leakage into organs, consistent with the pathophysiology of OHSS. These findings are also consistent with those reported earlier^42^, demonstrating that this experimental rat model is appropriate for the study of OHSS and that the results are translatable to humans with OHSS. Here, we also report an increase in the number of corpus lutea in OHSS-model rats compared to the control, indicative of an increase in the number of eggs released from mature follicles. The corpus luteum is a temporary ovarian structure; it is defined as the remnants of a mature follicle that has released an egg, and its function is dependent on whether the egg is subsequently fertilized. Interestingly, Tie1 knockdown in ovaries significantly decreased the weights of ovaries and the number of corpus lutea, as well as the VEGF level, suggesting that Tie1 may be a therapeutic target in cases of severe OHSS. It remains to be determined whether simultaneous knockdown of Tie1 and VEGF in ovaries can further reduce the ovarian weight to the control level. Further studies are needed to determine the importance of AKT phosphorylation in the development of OHSS, although any therapeutic entity capable of successfully treating moderate-to-severe cases of OHSS would require tissue-specific delivery.

EGR1, a transcription factor, has been reported to activate target genes whose proteins regulate a variety of cellular processes, including cell growth, inflammation, and wound repair^36,43^. Furthermore, EGR1 expression is regulated by growth factors, cytokines, and other factors. In granulosa cells, for example, the EGR1 level was upregulated by hCG, and these findings were linked to follicle maturation, ovulation, and luteinization^44^. Furthermore, there was a significant difference in the levels of EGR1 and VEGF in OHSS patients treated with cetrorelix, a GnRH antagonist, and untreated OHSS patients, with cetrorelix reducing the incidence of moderate and severe OHSS^45^. Other studies have reported an upregulation in EGR1 expression in several experimental models of vascular disease^35,36^. To examine the transcriptional regulation of Tie1, we constructed several constructs in which the EGR1 binding site was mutated and measured the activity of each promoter sequence using dual-luciferase assays. We found that the activity of the Tie1 promoter upon mutation of the EGR1 binding site within one region of the sequence was increased in SVOG cells treated with hCG but not upon mutation of the EGR1 binding site within two different regions. The interaction between the EGR1 transcription factor and the Tie1 promoter was confirmed by EGFR1 knockdown experiments. We found that the levels of Tie1, VEGF, and p-AKT were downregulated in EGFR1-silenced SVOG cells compared to the corresponding controls. Taken together, these findings reveal the regulatory mechanism responsible for the changes in these protein levels.

Here, we examined the roles of VEGF and Tie1 in the pathophysiology of OHSS and elucidated their mechanisms of action using in vitro and in vivo models of the disease. We found that VEGF and Tie1 levels were elevated in granulosa cells from patients with OHSS and rats with established OHSS, as well as in cells treated with a high dose of hCG, indicating that both proteins are involved in the pathophysiology of OHSS. However, further studies are needed to identify additional key proteins involved in these cellular events. We also established the relationship between VEGF and Ang-Tie signaling pathways, as determined by Tie1 knockdown experiments in vitro and in vivo, and showed that the mechanism of action also involved the PI3K/AKT cascade, indicating that the regulation of the ovarian vasculature is more complex than initially believed. Presently, it is not known whether the three signaling pathways interact in OHSS. Taken together, our results indicate that Tie1 may be a therapeutic target in cases of moderate-to-severe OHSS.
